# *Campylobacter jejuni* infection of conventionally colonized mice lacking nucleotide-oligomerization-domain-2

**DOI:** 10.1186/s13099-017-0155-3

**Published:** 2017-01-21

**Authors:** Stefan Bereswill, Ursula Grundmann, Marie E. Alutis, André Fischer, Markus M. Heimesaat

**Affiliations:** Department of Microbiology and Hygiene, Charité-University Medicine Berlin, CC5, Campus Benjamin Franklin, FEM, Garystr. 5, 14195 Berlin, Germany

**Keywords:** *Campylobacter jejuni*, Nucleotide-oligomerization-domain-2 (NOD2), In vivo infection, Intestinal microbiota, Colonization resistance, IL-23/IL-22/IL-18 axis, Pro-inflammatory immune responses, Bacterial translocation

## Abstract

**Background:**

The nucleotide-binding oligomerisaton protein 2 (NOD2) constitutes a pivotal sensor of bacterial muramyl dipeptide and assures expression of distinct antimicrobial peptides and mediators produced by enterocytes and immune cells directed against pathogens including *Campylobacter jejuni*. We here elucidated the role of NOD2 during murine *C. jejuni* infection in more detail.

**Results:**

Conventionally colonized NOD2 deficient (NOD2^−/−^) mice and corresponding wildtype (WT) counterparts were perorally infected with *C. jejuni* strain 81–176 on three consecutive days. The pathogen colonized both WT and NOD2^−/−^ mice only sporadically until day 14 post infection (p.i.). However, the slightly higher prevalence of *C. jejuni* in NOD2^−/−^ mice was accompanied by higher intestinal *Escherichia coli* loads known to facilitate *C. jejuni* colonization. Neither overt macroscopic (clinical) nor microscopic sequelae (such as colonic epithelial apoptosis) could be observed upon murine *C. jejuni* infection of either genotype. Innate immune responses were less distinctly induced in *C. jejuni* infected NOD2^−/−^ versus WT mice as indicated by lower colonic numbers of neutrophils in the former. Conversely, adaptive immune cell counts including T lymphocytes were higher in large intestines of NOD2^−/−^ as compared to WT mice that were paralleled by increased colonic IL-6 secretion and higher TNF and IL-18 mRNA expression levels in large intestines of the former. Only in NOD2^−/−^ mice, however, colonic IL-22 mRNA expression was down-regulated at day 14 p.i. Whereas viable commensal intestinal bacteria could exclusively be detected in mesenteric lymph nodes and livers of NOD2^−/−^ mice, bacterial translocation rates to kidneys and spleen were NOD2 independent. Notably, large intestinal mRNA expression levels of mucin-2, constituting a pivotal factor involved in epithelial barrier integrity, were comparable in naive and *C. jejuni* infected mice of either genotype.

**Conclusion:**

NOD2 is involved in the well-balanced regulation of innate and adaptive pro-inflammatory immune responses of conventional mice upon *C. jejuni* infection.

**Electronic supplementary material:**

The online version of this article (doi:10.1186/s13099-017-0155-3) contains supplementary material, which is available to authorized users.

## Background

Human *Campylobacter* infections are currently on the rise as indicated by increased prevalence and incidence rates in developed as well as developing countries [1, 2]. *C. jejuni* colonizes the intestinal tract of wild and domestic animals as a commensal, whereas humans usually become perorally infected by consumption of contaminated products derived from livestock animals or of surface water [3, 4]. Infected patients complain about gastroenteritis of varying degree ranging from mild malaise with watery diarrhea to severe ulcerative colitis with abdominal cramps, fever and inflammatory, bloody diarrhea [2, 5]. In the vast majority of cases, disease resolves spontaneously, whereas post-infectious sequelae affecting the nervous system (i.e. Guillain-Barré syndrome, Miller-Fisher syndrome and Bickerstaff encephalitis), the joints (i.e. reactive polyarthritis) or the intestinal tract (i.e. irritable bowel syndrome) might arise in rare cases with a latency of weeks to months [2, 5, 6]. Susceptibility of vertebrates to *Campylobacter* infections is highly depending on the host specific intestinal microbiota composition conferring physiological colonization resistance [7, 8]. Whereas conventionally colonized mice expell the pathogen from their intestinal tract within a few days following peroral *C. jejuni* challenge, modification of the murine intestinal microbiota by antibiotic treatment and reassociation with a human intestinal microbiota, for instance, results in stable pathogenic infection and, subsequently, distinct pro-inflammatory responses mimicking key features of human campylobacteriosis [7, 9] *C. jejuni* infection was further facilitated by pathophysiological conditions associated with increased intestinal commensal enterobacterial (i.e. *E. coli*) loads including acute and chronic intestinal inflammation and obesity [10, 11]. Also 3-weeks-old infant mice (immediately after weaning) harbored approximately two orders of magnitude higher intestinal commensal *E. coli* loads as compared to adult animals and were susceptible to *C. jejuni* infection, whereas the latter were not [12, 13]. In line with these results, artificial elevation of the intestinal *E. coli* loads in conventional adult mice by feeding a viable commensal *E. coli* strain via the drinking water was sufficient to override colonization resistance and resulted in stable pathogenic infecton upon peroral challenge [10].

The nucleotide-binding oligomerization domain (NOD) like receptors comprize intracellular pattern recognition receptors that regulate host immunity by sensing microbial products and damage-associated factors [14]. Among these, NOD2 is encoded by the *card15* gene and expressed at different levels by Paneth cells [15] and innate (dendritic cells, macrophages) as well as adaptive (i.e. T lymphocytes) immune cell populations [16–18]. Muramyl dipeptide (MDP) is a major constituent of bacterial peptidoglycan that is well-known for its adjuvant and immunomodulatory properties [19]. Furthermore, MDP from virtually all Gram-positive and Gram-negative bacteria can activate NOD2 conferring resistance against a plethora of bacterial species [14, 20–22]. Whether NOD2 is also capable of sensing other microbial structures or participates as a mere signaling partner is under current debate [23].

In the present study we addressed the role of NOD2 in *C. jejuni* infection of mice harboring a conventional intestinal microbiota and surveyed potential *C. jejuni* induced NOD2 dependent pro-inflammatory sequelae and bacterial translocation from the commensal intestinal microbiota to extra-intestinal compartments.

## Methods

### Ethics statement

All animal experiments were conducted according to the European Guidelines for animal welfare (2010/63/EU) with approval of the commission for animal experiments headed by the “Landesamt für Gesundheit und Soziales” (LaGeSo, Berlin, registration number G0135/10). Animal welfare was monitored twice daily by assessment of clinical conditions.

### Mice and *C. jejuni* infection

NOD2^−/−^ mice (in C57BL/6j background; initially obtained from The Jackson Laboratories, Bar Harbor, USA) and sex- and age-matched wildtype (WT) counterparts were bred, raised and maintained within the same specific pathogen free (SPF) unit in the Forschungseinrichtungen für Experimentelle Medizin (FEM, Charité-University Medicine Berlin). At the age of 3 months, female mice were perorally infected with 10^9^ colony forming units (CFU) of viable *C. jejuni* strain 81–176 in a volume of 0.3 mL phosphate buffered saline (PBS; Gibco, life technologies, Paisley, UK) on three consecutive days (days 0, 1 and 2) by gavage as described earlier [7].

### Sampling procedures

Mice were sacrificed at day 14 post infection (p.i.) by isofluran treatment (Abbott, Greifswald, Germany). Ex vivo biopsies from mesenteric lymph nodes (MLN), spleen, liver, kidney and the gastrointestinal tract (i.e. stomach, duodenum, ileum and colon) were asserved under sterile conditions. Colonic samples were collected in parallel for microbiological and immunological analyses. For immunohistological analyses, colonic ex vivo biopsies were immediately fixed in 5% formalin and embedded in paraffin.

### Quantitative analysis of bacterial colonization and translocation

For bacterial quantification within the gastrointestinal tract feces was taken over time p.i. and luminal samples were derived from stomach, duodenum, ileum and colon at necropsy (day 14 p.i.) and dissolved in sterile PBS. For determination of *C. jejuni* loads, serial dilutions were cultured on Columbia-Agar supplemented with 5% sheep blood and Karmali-Agar (both Oxoid, Wesel, Germany) for two days at 37 °C under microaerobic conditions using CampyGen gas packs (Oxoid). For quantification of *E. coli*, serial dilutions were cultured on Columbia-Agar supplemented with 5% sheep blood and Mac Conkey Agar (both Oxoid) in aerobic atmosphere for two days at 37 °C.

Translocation of commensal intestinal bacteria to extra-intestinal compartments was quantitatively assessed in respective organ homogenates under aerobic, microaerobic and obligate anaerobic conditions as described earlier [24–26].

The respective weights of fecal or tissue samples were determined by the difference of the sample weights before and after asservation. The detection limit of viable pathogens was ≈100 CFU per g.

### Immunohistochemistry

Five µm thin paraffin sections of colonic ex vivo biopsies were subjected to in situ immunohistochemical analysis as described previously [27–29]. In brief, primary antibodies against cleaved caspase-3 (Asp175, Cell Signaling, Boston, MA, USA, 1:200), Ki67 (TEC3; Dako, Glostrup, Denmark; 1:100), CD3 (#N1580; Dako; 1:10), FOXP3 (FJK-16 s; eBioscience, San Diego, CA, USA; 1:100), B220 (eBioscience; 1:200) and myeloperoxidase (MPO-7, # A0398; Dako; 1:500) were used to assess apoptotic cells, proliferating/regenerating cells, T lymphocytes, regulatory T cells (Treg), B lymphocytes and neutrophils, respectively. The average number of positively stained cells within at least six high power fields (HPF, 0.287 mm^2^; 400× magnification) were determined by an independent and blinded investigator.

### Cytokine detection in supernatants of colonic ex vivo biopsies

Colonic ex vivo biopsies were cut longitudinally and washed in PBS. Strips of approximately 1 cm^2^ large intestinal tissues were placed in 24-flat-bottom well culture plates (Nunc, Wiesbaden, Germany) containing 500 μL serum-free RPMI 1640 medium (Gibco) supplemented with penicillin (100 U/mL) and streptomycin (100 µg/mL; PAA Laboratories, Cölbe, Germany). After 18 h at 37 °C culture supernatants were tested for IL-6 and IL-10 by the Mouse Inflammation Cytometric Bead Assay (CBA; BD Biosciences, Heidelberg, Germany) on a BD FACSCanto II flow cytometer (BD Biosciences) as described previously [30].

### Real-time PCR

RNA was isolated from snap frozen colonic ex vivo biopsies, reverse transcribed and analyzed as described previously [31]. Murine TNF, IFN-γ, IL-23p19, IL-22, IL-18 and mucin-2 mRNA expression levels were detected by real-time PCR with specific primers and quantified by analysis with the Light Cycler Data Analysis Software (Roche Life Science, Mannheim, Germany). The mRNA of the housekeeping gene for hypoxanthine-phosphoribosyltransferase (HPRT) was used as reference, and the mRNA expression levels of the individual genes were normalized to the lowest measured value and expressed as fold expression (Arbitrary Units).

### Statistical analysis

Medians and levels of significance were determined using the Mann–Whitney U test (GraphPad Prism v5, La Jolla, CA, USA) as indicated. Two-sided probability (p) values ≤0.05 were considered significant.

## Results

### Pathogenic colonization properties in *C. jejuni* infected conventionally colonized NOD2^−/−^ mice

**Fig. 1:**
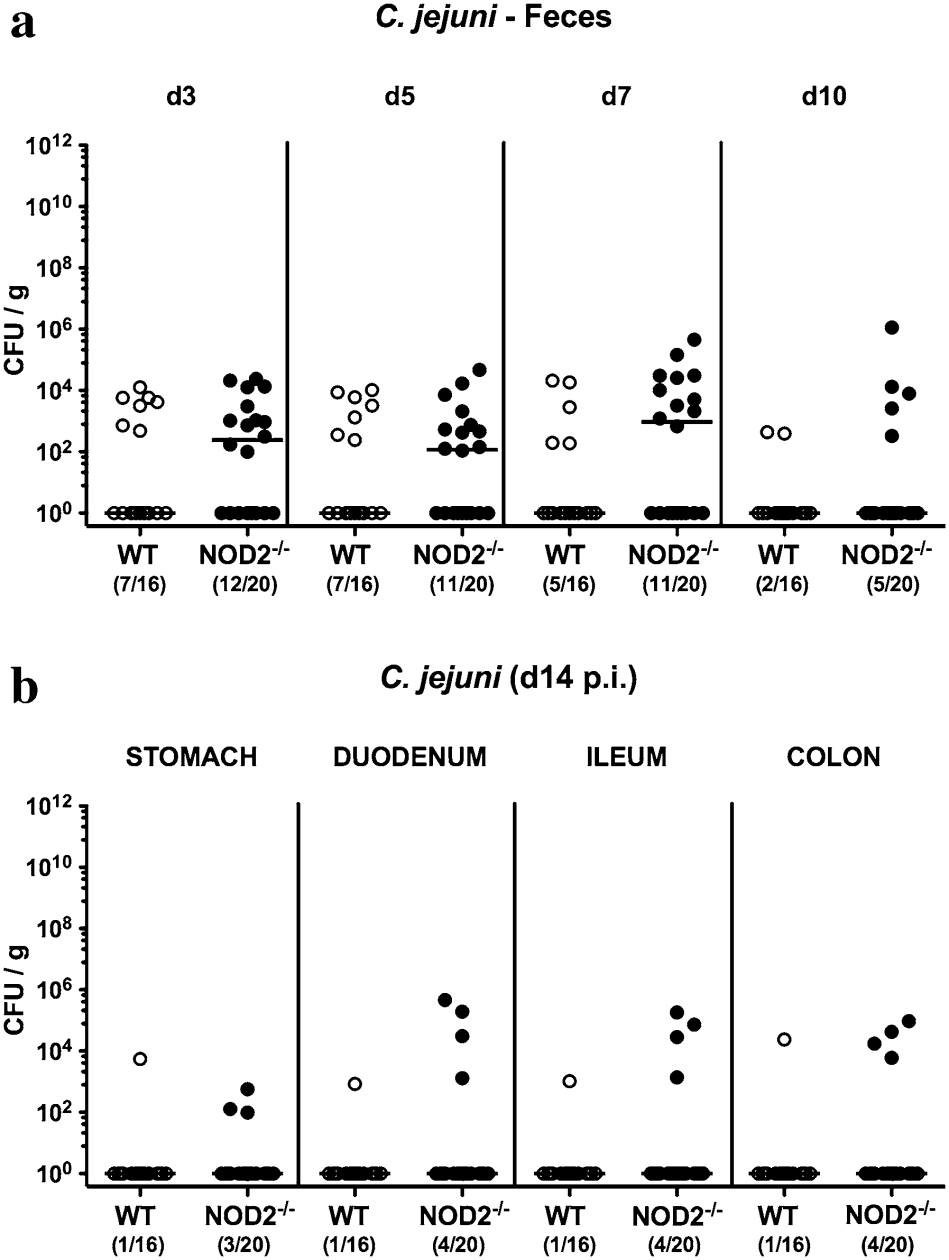
Gastrointestinal *C. jejuni* loads in perorally infected conventionally colonized NOD2^−/−^ mice. Wildtype (WT; *white circles*) and NOD2^−/−^ mice (*black circles*) were perorally infected with *C. jejuni* strain 81–176 on three consecutive days (d0, 1 and 2). **a** Pathogenic colonization densities were assessed in fecal samples (CFU, colony forming units per gram) over time post infection (p.i.). **b** At day of necrosy (d14 p.i.) *C. jejuni* loads were determined in luminal samples derived from distinct parts of the gastrointestinal tract. Medians (*black bars*) are indicated and numbers of mice harboring *C. jejuni* strain 81-176 out of the total number of analyzed animals are given in parentheses. Data were pooled from four independent experiments

In order to investigate the role of NOD2 during *C. jejuni* infection of mice harboring a conventional microbiota, NOD2^−/−^ and corresponding WT control mice were perorally infected with 10^9^ CFU *C. jejuni* strain 81–176 on three consecutive days (days 0, 1 and 2). Within 24 h following the latest infection viable *C. jejuni* could be detected in 60.0% of fecal samples derived from NOD2^−/−^ mice with low median loads of approximately 10^2^ CFU per g feces only (Fig. 1a; Additional file 1: Figure S1). More than half of WT mice, however, had expelled the pathogen from their intestinal tract in the meantime, given that *C. jejuni* could be isolated in only 43.8% of feces derived from WT mice with median loads below the detection limit (Fig. 1a; Additional file 1: Figure S1). Later during the course of infection pathogenic loads further declined in mice of either genotype with 20.0 and 6.3% of NOD2^−/−^ and WT mice, respectively, harboring *C. jejuni* in their feces at day 14 p.i. (Fig. 1; Additional file 1: Figure S1). Despite higher pathogenic positivity rates in NOD2^−/−^ vs WT mice, intestinal *C. jejuni* loads did not differ at defined time points p.i. (n.s; Fig. 1a). At day of necropsy (i.e. day 14 p.i.), *C. jejuni* could be isolated from the stomach, proximal and distal small intestines as well as the colon in single cases only (n.s.; Fig. 1b).

**Fig. 2:**
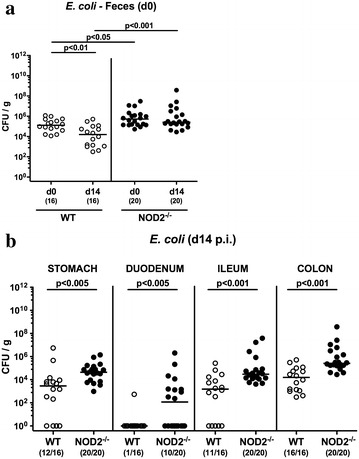
Commensal intestinal *E. coli* loads in *C. jejuni* infected conventionally colonized NOD2^−/−^ mice. Wildtype (WT; *white circles*) and NOD2^−/−^ mice (*black circles*) were perorally infected with *C. jejuni* strain 81–176 on three consecutive days (d0, 1 and 2). Commensal *E. coli* loads were compared (**a**) in feces immediately before infection (d0) and 14 days thereafter and determined (**b**) in luminal samples derived from the gastrointestinal tract at day 14 following *C. jejuni* challenge. Medians (*black bars*) and levels of significance (p values) determined by Mann–Whitney U test are indicated. Numbers of mice harboring commensal *E. coli* out of the total number of analyzed animals are given in parentheses. Data were pooled from four independent experiments

Given that elevated intestinal loads of commensal *E. coli* facilitate *C. jejuni* colonization [10], we next assessed fecal *E. coli* densities in NOD2^−/−^ and WT mice immediately before and 14 days after *C. jejuni* infection. Naive and infected NOD2^−/−^ mice exhibited approximately 0.5 and 1.0 log, respectively, higher fecal *E. coli* densities as compared to respective WT counterparts (p < 0.05 and p < 0.001, respectively; Fig. 2a). Within 14 days following pathogenic challenge, *E. coli* loads declined by less than one order of magnitude in fecal samples of WT mice (p < 0.01; Fig. 2a). At day of necropsy, *E. coli* loads were up to two orders of magnitude higher in luminal samples taken from the stomach, duodenum, ileum and colon of NOD2^−/−^ as compared to WT mice (p < 0.005–0.001; Fig. 2b). Taken together, NOD2 deficiency did not impact gastrointestinal colonization properties of *C. jejuni* in conventionally colonized mice. Slightly higher percentages of *C. jejuni* infected NOD2^−/−^ mice were associated with higher commensal intestinal *E. coli* loads as compared to WT controls.

### Microscopic sequelae and pro-inflammatory cell responses upon *C. jejuni* infection of conventional NOD2^−/−^ mice

**Fig. 3:**
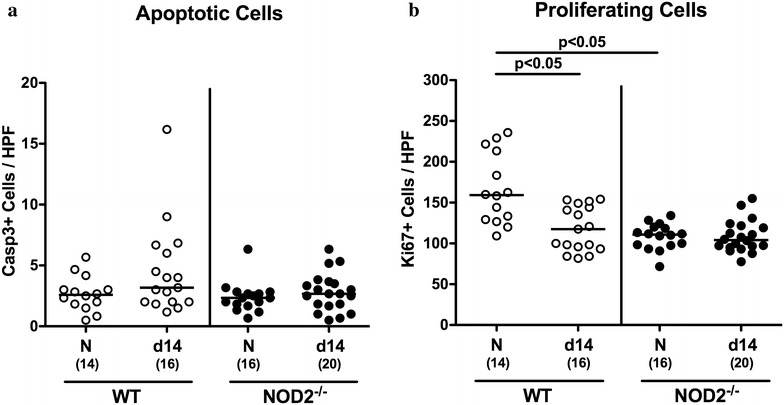
Apoptotic and proliferating cells in the colonic epithelium of *C. jejuni* infected conventionally colonized NOD2^−/−^ mice. Wildtype (WT; *white circles*) and NOD2^−/−^ mice (*black circles*) were perorally infected with *C. jejuni* strain 81–176 on three consecutive days (d0, 1 and 2). The average number of colonic (**a**) apoptotic cells (positive for caspase-3, Casp3) and (**b**) proliferating cells (positive for Ki67) from six high power fields (HPF, 400× magnification) per animal was determined microscopically in immunohistochemically stained colonic paraffin sections at day 14 following *C. jejuni* infection. Naive (N) mice served as uninfected controls. Medians (*black bars*), levels of significance (p values) determined by Mann–Whitney U test and numbers of analyzed animals (in *parentheses*) are indicated. Data were pooled from four independent experiments

Following *C. jejuni* infection mice of either genotype were virtually uncompromized from their clinical aspect. Given that apoptosis comprizes a valuable marker for the histopathological grading of intestinal inflammation and is a key feature of campylobacteriosis [7], we next stained colonic paraffin sections of infected mice against caspase-3 by in situ immunohistochemistry. As for the macroscopic aspect, no changes in numbers of apoptotic cells in colonic epithelia could be observed at day 14 following *C. jejuni* infection of mice irrespective of their genotype (n.s.; Fig. 3a). We also performed in situ immunhistochemical stainings of colonic sections against the proliferation marker Ki67. Interestingly, naive NOD2^−/−^ mice exhibited approximately one-third lower numbers of Ki67+ colonic epithelial cells as compared to WT controls (p < 0.05; Fig. 3b), whereas large intestinal proliferating cell numbers declined by more than one-third in WT, but not NOD2^−/−^ mice until day 14 following *C. jejuni* infection (p < 0.05; Fig. 3b).

**Fig. 4:**
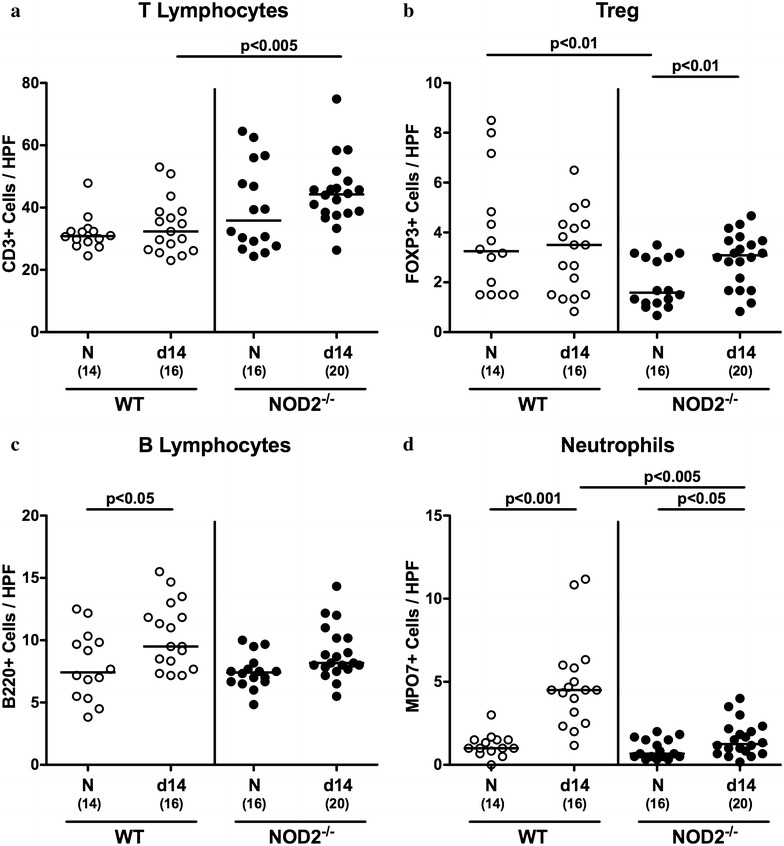
Colonic immune cell responses in *C. jejuni* infected conventionally colonized NOD2^−/−^ mice. Wildtype (WT; white *circles*) and NOD2^−/−^ mice (*black circles*) were perorally infected with *C. jejuni* strain 81–176 on three consecutive days (d0, 1 and 2). The average number of colonic **a** T lymphocytes (positive for CD3) **b** regulatory T cells (Treg, positive for FOXP3) **c** B lymphocytes (positive for B220) and **d** neutrophils (positive for MPO7) from six high power fields (HPF, 400× magnification) per animal was determined microscopically in immunohistochemically stained colonic paraffin sections at day 14 following *C. jejuni* infection. Naive (N) mice served as uninfected controls. Medians (*black bars*), levels of significance (p values) determined by Mann–Whitney U test and numbers of analyzed animals (in *parentheses*) are indicated. Data were pooled from four independent experiments

Given that recruitment of pro-inflammatory immune cells to the site of infection is a hall mark of intestinal inflammation including campylobacteriosis [7], we next quantitatively assessed distinct innate and adaptive immune cell populations in the large intestinal mucosa and lamina propria of infected mice applying in situ immunohistochemistry. At day 14 p.i., colonic T lymphocyte numbers were higher in NOD2^−/−^ as compared to WT mice (p < 0.005; Fig. 4a). Following *C. jejuni* infection Treg numbers increased in the large intestines of NOD2^−/−^, but not WT mice (p < 0.01; Fig. 4b). Notably, under naive conditions colonic Treg numbers were lower in NOD2^−/−^ as compared to WT mice (p < 0.01; Fig. 4b). Whereas upon *C. jejuni* infection colonic B lymphocytes were elevated in WT mice only (p < 0.05; Fig. 4c), neutrophil numbers increased in the large intestinal mucosa and lamina propria of both WT and NOD2^−/−^ at day 14 p.i. (p < 0.001 and p < 0.05, respectively; Fig. 4d), but to a lesser extent in the latter (p < 0.005; Fig. 4d).

### Pro- and anti-inflammatory cytokine responses upon *C. jejuni* infection of conventional NOD2^−/−^ mice

**Fig. 5:**
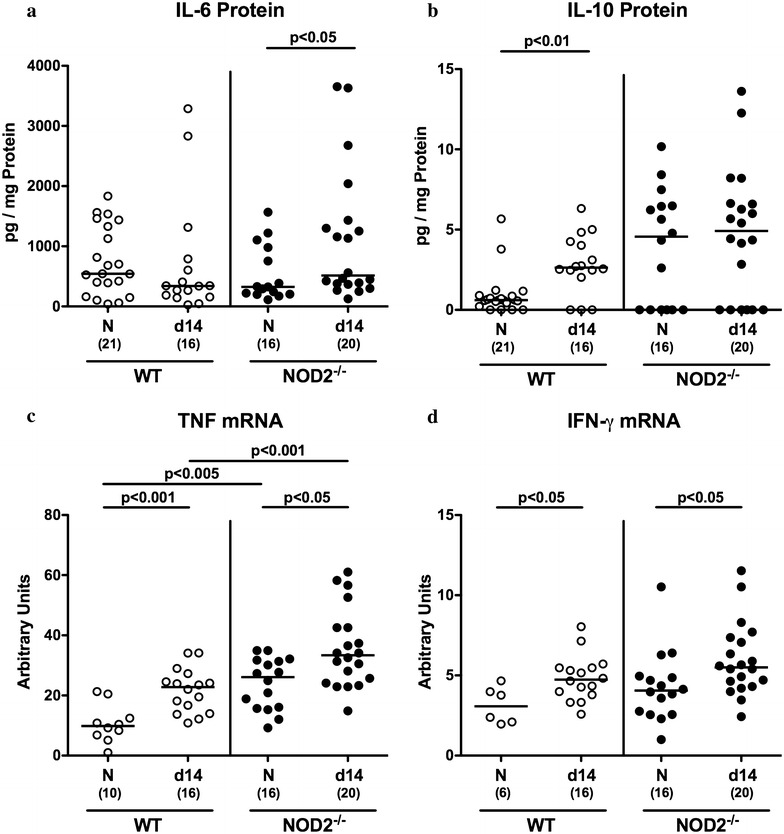
Colonic cytokines in *C. jejuni* infected conventionally colonized NOD2^−/−^ mice. Wildtype (WT; *white circles*) and NOD2^−/−^ mice (*black circles*) were perorally infected with *C. jejuni* strain 81–176 on three consecutive days (d0, 1 and 2). **a** IL-6 and **b** IL-10 protein concentrations were measured in colonic ex vivo biopsies at day 14 post infection. In additon, large intestinal **c** TNF and **d** IFN-γ mRNA expression levels were determined by real time PCR and expressed as arbitrary units (fold expression). Naive (N) mice served as uninfected controls. Medians (*black bars*), levels of significance (p value) determined by Mann–Whitney U test and numbers of analyzed animals (in *parentheses*) are indicated. Data were pooled from four independent experiments

We next measured secretion of the pro-inflammatory cytokine IL-6 and the anti-inflammatory mediator IL-10 in colonic ex vivo biopsies. Whereas colonic IL-6 levels increased in *C. jejuni* infected NOD2^−/−^ mice only (p < 0.05; Fig. 5a), IL-10 concentrations were higher in the large intestines of WT (p < 0.01; Fig. 5b), but not NOD2^−/−^ mice at day 14 p.i. as compared to naive controls. We further analyzed colonic expression levels of the pro-inflammatory cytokines TNF and IFN-γ. At day 14 p.i., increased mRNA levels of either pro-inflammatory mediator could be determined in colonic ex vivo biopsies of both NOD2^−/−^ and WT mice (p < 0.05 – 0.001; Fig. 5c, d). Interestingly, the *C. jejuni* induced TNF up-regulation was more pronounced in infected NOD2^−/−^ as compared to WT mice (p < 0.001; Fig. 5c). Moreover, also in the naive state, TNF mRNA levels were higher in NOD2^−/−^ than WT mice (p < 0.005; Fig. 5c).

**Fig. 6:**
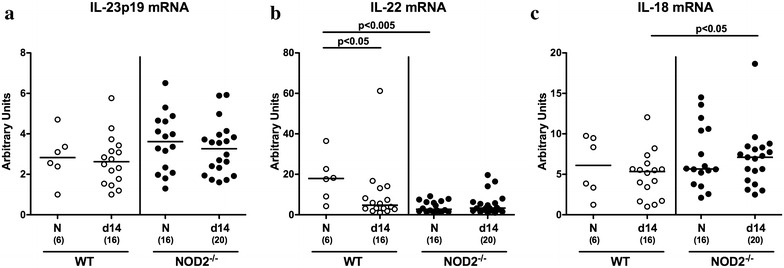
Colonic expression of IL-23p19, IL-22 and IL-18 mRNA in *C. jejuni* infected conventionally colonized NOD2^−/−^ mice. Wildtype (WT; *white circles*) and NOD2^−/−^ mice (*black circles*) were perorally infected with *C. jejuni* strain 81–176 on three consecutive days (d0, 1, and 2). **a** IL-23p19 **b** IL-22 and **c** IL-18 mRNA expression levels were determined in colonic ex vivo biopsies at day 14 post infection by real time PCR and expressed as arbitrary units (fold expression). Naive (N) mice served as uninfected controls. Medians (*black bars*), levels of significance (p value) determined by Mann–Whitney U test and numbers of analyzed animals (in *parentheses*) are indicated. Data were pooled from four independent experiments

Our group could recently show that the IL-23/IL-22/IL-18 axis is involved in mediating murine *C. jejuni* infection [32–36]. We therefore determined potential NOD2 dependent mRNA expression of respective mediators during murine *C. jejuni* infection. Whereas colonic IL-23p19 mRNA levels did not differ in naive and infected mice of either genotype (n.s.; Fig. 6a), IL-22 mRNA was down-regulated in large intestines of infected WT mice only (p < 0.05; Fig. 5b), whereas colonic IL-18 mRNA expression levels were higher in infected NOD2^−/−^ as compared to WT mice at day 14 p.i. (p < 0.05; Fig. 5c). Notably, IL-22 mRNA levels were lower in naive NOD2^−/−^ than WT mice (p < 0.005; Fig. 5b). Hence, large intestinal IL-6 secretion as well as TNF and IL-18 mRNA levels were higher in NOD2^−/−^ as compared to WT mice upon pathogenic infection, whereas *C. jejuni* induced colonic IL-22 down-regulation occurred in WT mice only.

### Translocation of intestinal commensal species to extra-intestinal compartments following *C. jejuni* infection of conventional NOD2^−/−^ mice

**Fig. 7:**
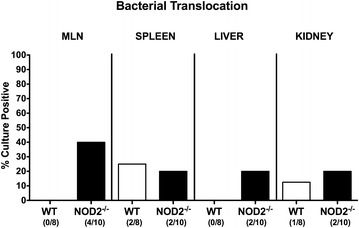
Pathogenic translocation in *C. jejuni* infected conventionally colonized NOD2^−/−^ mice. Wildtype (WT; *white bars*) and NOD2^−/−^ mice (*black bars*) were perorally infected with *C. jejuni* strain 81–176 on three consecutive days (d0, 1 and 2). Translocation of viable pathogens were assessed in ex vivo biopsies derived from mesenteric lymph nodes (MLN), spleen, liver and kidney at day 14 post infection by culture. Relative abundances of viable pathogens in respective compartments are shown (*bars*; in %) and numbers of mice harboring *C. jejuni* out of the total number of analyzed animals (in *parentheses*) are indicated. Data shown are representative for two independent experiments

We next addressed whether *C. jejuni* infection was associated with translocation of viable bacteria originating from the commensal intestinal microbiota to MLN and extra-intestinal compartments. Notably, in naive mice all organ homogenates were free of viable bacteria (not shown). Whereas no bacteria could be detected in MLN and liver homogenates of WT mice at day 14 p.i., commensal intestinal bacterial species were isolated from 40.0% of MLN and 20.0% of liver samples derived from *C. jejuni* infected NOD2^−/−^ mice by direct plating (Fig. 7). Bacterial translocation rates into spleens were 25.0 and 20.0% in WT and NOD2^−/−^ mice, respectively, whereas commensal bacterial species could be isolated in 12.5 and 20.0% of kidneys taken from WT and NOD2^−/−^ mice, respectively, at day 14 p.i. (Fig. 7). In culture-positive cases, intestinal commensals such as *E. coli*, *Enterococcus* spp. and/or *Lactobacillus* spp. could be isolated.

Mucin-2 (MUC-2) constitutes a pivotal component of the mucus layer covering the intestinal epithelium and thereby combats bacterial infections and maintains epithelial barrier integrity [37, 38]. We therefore raised the question whether mucin-2 expression was affected in NOD2^−/−^ mice thereby facilitating bacterial translocation. Neither under naive conditions nor at day 14 following *C. jejuni* infection, however, mucin-2 mRNA expression levels differed in the colon of NOD2^−/−^ and WT mice (n.s.; Additional file 2: Figure S2).

## Discussion

The well-orchestrated interaction of distinct immune cells, pattern recognition receptors and evolving signaling pathways is pivotal to prevent the vertebrate host from infections with invading pathogens including *C. jejuni*. In the present study we investigated the impact of the bacterial MDP sensor NOD2 during *C. jejuni* infection of conventionally colonized mice. Despite peroral challenge with high pathogenic loads even on three consecutive days, both WT and NOD2^−/−^ could be colonized only sporadically until day 14 p.i., which is due to the physiological colonization resistance mediated by the distinct murine microbiota composition [7, 9, 12, 13, 34, 39, 40]. During the entire time course following infection pathogenic positivity rates of fecal samples were slightly higher in NOD2^−/−^ as compared to WT mice. This might be explained by concomitant slightly higher loads of commensal *E. coli* (between one and two orders of magnitude) within the gastrointestinal lumen known to facilitate *C. jejuni* colonization [7, 8, 10, 12, 13, 34, 39, 40]. In addition, NOD2 deficiency is associated with defective expression of antimicrobial peptides resulting in compromized pathogenic clearance by the host [41, 42].

Several studies further revealed the importance of NOD2 in sensing and elimination of pathogens, given that NOD2^−/−^ mice have been shown to be more susceptible to infection with *Salmonella Typhimurium* [43] or *Listeria monocytogenes* [42]. A previous elegant in vivo study revealed that NOD2 is essential for the control of campylobacteriosis in antibiotics-treated mice lacking IL-10 [44]. Interestingly, *C. jejuni* induced colitis was more pronounced in NOD2^−/−^ IL-10^−/−^ mice as compared to IL-10^−/−^ mice. The authors further demonstrated that NOD2 was essential for nitric oxide production in peritoneal macrophages. Based on the finding that nitroprusside attenuated murine campylobacteriosis the authors concluded that NOD2 is essential for pathogen control by bactericidal responses involving nitric oxide [44]. Differences regarding disease outcomes when compared to our report are due to substantial differences regarding the applied animal models. Whereas Su and colleagues had pretreated IL-10^−/−^ mice with broad-spectrum antibiotics prior *C. jejuni* infection, we here investigated WT animals harboring a conventional microbiota.

In line with our previous reports, also in the present study *C. jejuni* infected conventionally colonized mice of either genotype were neither clinically compromized (by wasting or abundance of bloody diarrhea, for instance), nor could microscopic sequelae such as colonic epithelial apoptosis be observed at necropsy [7–9, 30]. One could assume that lack of overt pathological responses might be due to successful clearance of the enteropathogen during the course of infection by the host. As shown earlier, however, *C. jejuni* does not necessarily need to permanently establish witin the intestinal tract to evoke pro-inflammatory host responses [30, 32, 36, 45]. It is rather the initial hit of the enteropathogenic infection that tips the balance towards immunopathological host responses [30]. In support of this hypothesis, *C. jejuni* induced large intestinal immune cell responses could, in fact, be observed also in the present study as indicated by elevated Treg numbers in infected NOD2^−/−^ mice only, whereas B lymphocytes increased exclusively in the large intestines of WT mice. In addition, innate immune responses were more pronounced in NOD2 deficient mice as indicated by an increased influx of neutrophils into the colonic mucosa and lamina propria following *C. jejuni* infection that was more pronounced in NOD2^−/−^ as compared to WT mice and paralleled by higher levels of pro-inflammatory cytokines including IL-6 and TNF. Conversely, colonic concentrations of the anti-inflammatory cytokine IL-10 was elevated in WT, but not NOD2^−/−^ mice at day 14 p.i., further supporting the overall more pronounced *C. jejuni* induced pro-inflammatory host responses upon NOD2 deficiency.

Very recently our group has highlighted the importance of the IL-23/IL-22/IL-18 axis in campylobacteriosis [30, 32, 34, 36]. In infected secondary abiotic WT mice, for instance, colonic IL-23p19, IL-22 and IL-18 were all upregulated [36], whereas large intestinal IL-22 mRNA levels were shown to be increased in infected IL-10^−/−^ mice [46]. As member of the IL-10 cytokine family, IL-22 can exert dichotomous modes of action depending on the respective tissue (i.e. compartment of the intestinal tract) and the surrounding cytokine milieu [30, 47, 48]. Whereas in the small intestines IL-22 exerts pro-inflammatory properties as shown in murine *Toxoplasma gondii* induced ileitis [31, 49, 50], IL-22 has anti-inflammatory functions in the colon [48]. Interestingly, in the present study basal IL-22 mRNA levels were lower in large intestines of NOD2^−/−^ as compared to WT mice. Given that IL-22 was shown to be effective in antimicrobial host defence against *C. jejuni* [51], down-regulated basal IL-22 levels might explain slightly higher fecal pathogenic positivity rates in NOD2^−/−^ as compared to WT mice shown here. *C. jejuni* infection, however, resulted in down-regulation of colonic IL-22 expression in WT animals only, whereas neither IL-23p19 (as well-known master regulator of mucosal immune responses [52]) nor IL-18 (amplifying IL-22 production during intestinal inflammation [50]), were affected upon infection, which is well in line with our very recent results derived in *C. jejuni* infected conventional mice as well [30].

Epithelial barrier integrity is of utmost importance for limiting bacterial/pathogenic spread from the intestinal compartment to extra-intestinal including systemic compartments with potentially fatal consequences for the host [53]. Whereas in the present study bacterial translocation could not be observed in any naive mice of both genotypes, viable commensal intestinal species such as *E. coli*, enterococci and/or lactobacilli could be exclusively detected in MLN and livers of NOD2^−/−^ mice, whereas bacterial translocation rates to kidney and spleen were rather comparable. Given that mucins including MUC-2 are pivotal components of the viscous mucous layer preserving epithelial barrier function by protecting the underlying mucosal epithelial layer not only from invading pathogens, but also from translocating intestinal commensals [53, 54], we analyzed MUC-2 expression in the colon of NOD2^−/−^ and WT mice before and 14 days after *C. jejuni* infection. We could, however, not observe significant differences in colonic MUC-2 expression that might explain the observed differences in bacterial translocation rates. One needs to take into consideration that epithelial barrier function is warranted by a complex interaction of many independent factors (with MUC-2 mRNA expression only one amongst plenty) [55].

We conclude that NOD2 is involved in the well-balanced regulation of innate and adaptive pro-inflammatory immune responses of conventional mice upon *C. jejuni* infection. Future studies are needed to unravel the underlying molecular mechanisms in more detail.

## Additional files

**Additional file 1: Figure S1.** Kinetic of intestinal *C. jejuni* loads in perorally infected conventionally colonized NOD2^-/-^ mice. **(A)** Wildtype (WT; white circles) and **(B)** NOD2^-/-^ mice (black circles) were perorally infected with *C. jejuni* strain 81-176 on three consecutive days (d0, 1 and 2). Pathogenic colonization densities were assessed in fecal samples (CFU, colony forming units per gram) over time post infection as indicated by culture. Medians (black bars) and levels of significance (p values) determined by Mann-Whitney U test are indicated. Numbers of mice harboring *C. jejuni* out of the total number of analyzed animals are given in parentheses. Data were pooled from four independent experiments.

**Additional file 2: Figure S2.** Colonic mucin-2 mRNA expression levels in *C. jejuni* infected conventionally colonized NOD2^-/-^ mice. Wildtype (WT; white circles) and NOD2^-/-^ mice (black circles) were perorally infected with *C. jejuni* strain 81-176 on three consecutive days (d0, 1 and 2). Mucin-2 (MUC-2) mRNA expression levels were determined in colonic *ex vivo* biopsies at day 14 post infection by Real Time PCR and expressed in Arbitrary Units (fold expression). Naive (N) mice served as uninfected controls. Medians (black bars) and numbers of analyzed animals (in parentheses) are indicated. Data were pooled from four independent experiments.

## Abbreviations

NOD2: nucleotide-binding oligomerization domain protein 2
MDP: muramyl dipeptide
WT: wildtype
SPF: special pathogen-free
CFU: colony forming units
PBS: phosphate buffered saline
p.i.: post infection
MLN: mesenteric lymph nodes
Treg: regulatory T cells
MPO: myeloperoxidase
HPF: high power field
HPRT: hypoxanthine-phosphoribosyltransferase
MUC-2: mucin-2
PCR: polymerase chain reaction
