# Supplementary figures and images for: Campylobacter jejuni infection of conventionally colonized mice lacking nucleotide-oligomerization-domain-2
Source: Gut Pathog. 2017 Jan 21;9:5. doi: 10.1186/s13099-017-0155-3 (PMC5251327; doi:10.1186/s13099-017-0155-3)

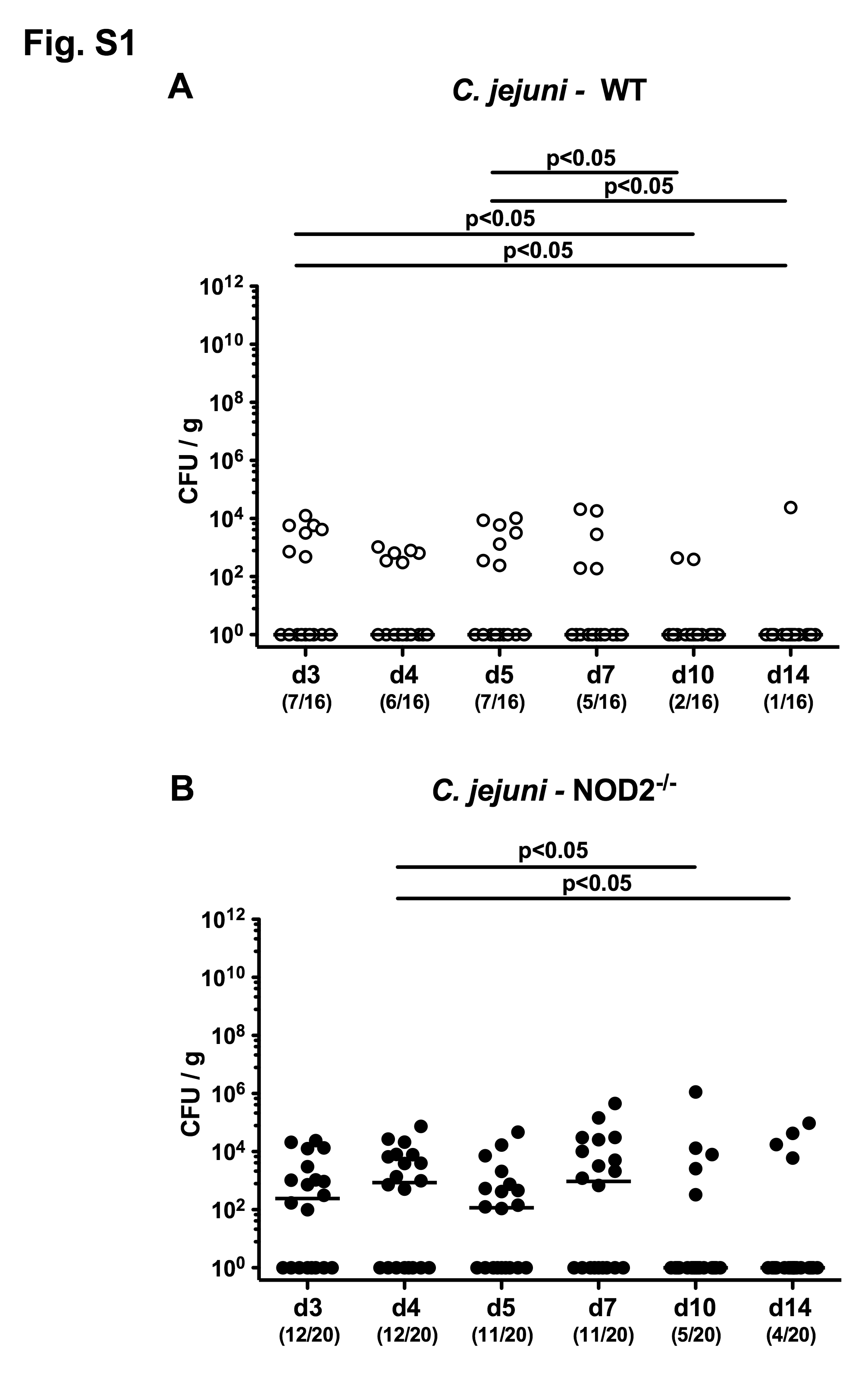

Supplement: Supplementary file 1 — Additional file 1: Figure S1. Kinetic of intestinal C. jejuni loads in perorally infected conventionally colonized NOD2-/- mice. (A) Wildtype (WT; white circles) and (B) NOD2-/- mice (black circles) were perorally infected with C. jejuni strain 81-176 on three consecutive days (d0, 1 and 2). Pathogenic colonization densities were assessed in fecal samples (CFU, colony forming units per gram) over time post infection as indicated by culture. Medians (black bars) and levels of significance (p values) determined by Mann-Whitney U test are indicated. Numbers of mice harboring C. jejuni out of the total number of analyzed animals are given in parentheses. Data were pooled from four independent experiments. [file 13099_2017_155_MOESM1_ESM.tiff]

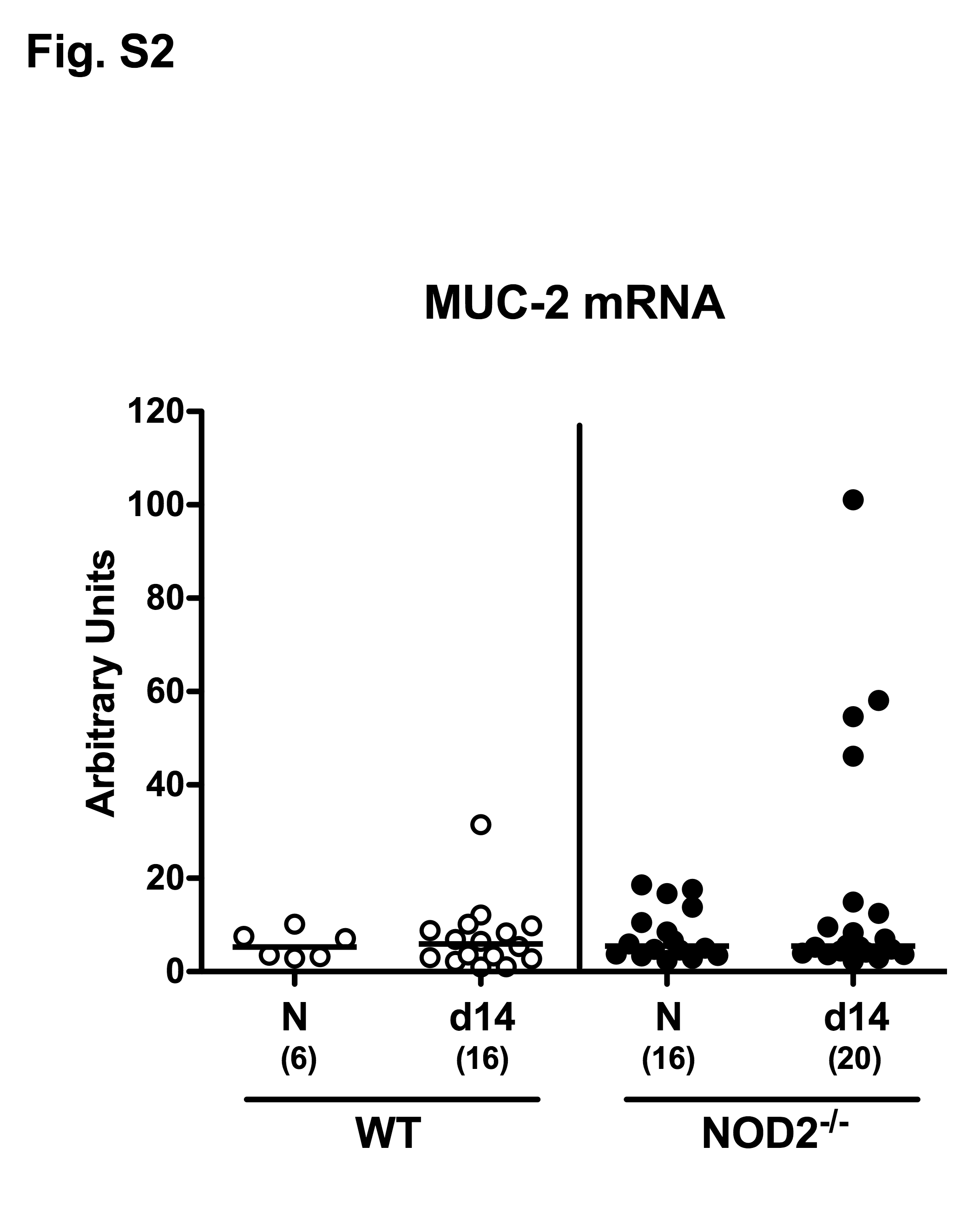

Supplement: Supplementary file 2 — Additional file 2: Figure S2. Colonic mucin-2 mRNA expression levels in C. jejuni infected conventionally colonized NOD2-/- mice. Wildtype (WT; white circles) and NOD2-/- mice (black circles) were perorally infected with C. jejuni strain 81-176 on three consecutive days (d0, 1 and 2). Mucin-2 (MUC-2) mRNA expression levels were determined in colonic ex vivo biopsies at day 14 post infection by Real Time PCR and expressed in Arbitrary Units (fold expression). Naive (N) mice served as uninfected controls. Medians (black bars) and numbers of analyzed animals (in parentheses) are indicated. Data were pooled from four independent experiments. [file 13099_2017_155_MOESM2_ESM.tiff]
